# Association of insulin resistance and coronary artery remodeling: an intravascular ultrasound study

**DOI:** 10.1186/s12933-015-0238-8

**Published:** 2015-06-06

**Authors:** Sang-Hoon Kim, Jae-Youn Moon, Yeong Min Lim, Kyung Ho Kim, Woo-In Yang, Jung-Hoon Sung, Seung Min Yoo, In Jai Kim, Sang-Wook Lim, Dong-Hun Cha, Seung-Yun Cho

**Affiliations:** Department of Cardiology, CHA Bundang Medical Center, CHA University, Seongnam, South Korea; Department of Diagnostic Radiology, CHA Bundang Medical Center, CHA University, Seongnam, South Korea

**Keywords:** Insulin resistance, Metabolic syndrome, Coronary artery remodeling, Intravascular ultrasound

## Abstract

**Background:**

There are few studies that investigated the correlation between insulin resistance (IR) and the coronary artery remodeling. The aim of the study is to investigate the association of IR measured by homeostasis model assessment of insulin resistance (HOMA-IR) and coronary artery remodeling evaluated by intravascular ultrasound (IVUS).

**Methods:**

A total of 298 consecutive patients who received percutaneous coronary interventions under IVUS guidance were retrospectively enrolled. The value of HOMA-IR more than 2.5 was considered as IR positive. Metabolic syndrome was classified according to NCEP ATP III guidelines. The remodeling index was defined as the ratio of the external elastic membrane (EEM) area at the lesion site to the EEM area at the proximal reference site.

**Results:**

A total of 369 lesions were analyzed (161 lesions in HOMA-IR positive and 208 lesions in HOMA-IR negative). Remodeling index was significantly higher in the HOMA-IR positive group compared with the negative group (HOMA-IR positive vs. negative: 1.074 ± 0.109 vs. 1.042 ± 0.131, *p* = 0.013). There was a significant positive correlation between remodeling index and HOMA-IR (*p* = 0.010). Analysis of HOMA-IR according to remodeling groups showed increasing tendency of HOMA-IR, and it was statistically significant (*p* = 0.045). Multivariate analysis revealed that only HOMA-IR was an independent predictor of remodeling index (*r* = 0.166, *p* = 0.018).

**Conclusion:**

Increased IR estimated by HOMA-IR was significantly associated with a higher remodeling index and positive coronary artery remodeling.

## Background

Coronary artery remodeling is changes in the external elastic membrane (EEM), typically occurred in response to atherosclerotic plaque accumulation. Positive remodeling (PR) refers to a larger EEM area at a lesion site than the adjacent reference site, and negative remodeling (NR) refers to a smaller EEM area than the adjacent reference site. Several studies have demonstrated that PR is more predominant in patients with acute coronary syndrome (ACS) comparing to patients with stable angina pectoris [1–3]. Previous study have reported that a PR lesion has higher lipid contents and a macrophage count, both markers of plaque vulnerability in a necropsy study [4]. It is also known that NR is a common finding in diabetic patients and is associated with several factors like smoking, hypertension and plasma homocysteine levels [5–7]. Advanced glycation end-products are known as key substances involved in the negative remodeling associated with diabetes. Smoking causes endothelial dysfunction, increased oxidative stress, and decreased nitric oxide synthesis leading to inward remodeling [8].

Insulin resistance (IR) is mediated by the interaction of a person’s genetic characteristics and acquired pathophysiologic insults by personal lifestyle. Molecular studies demonstrated that IR has a key role in every stage of atherosclerosis from the initiation to the clinically significant progression of plaques [9]. Previous studies reported that IR was associated with the coronary artery calcium score and the severity of coronary artery disease [10–12]. However, there are few reports that investigated the correlation of IR or metabolic syndrome (MetS) with the coronary artery remodeling. In this study, we investigated the association between IR measured by homeostasis model assessment of insulin resistance (HOMA-IR) and coronary artery remodeling by intravascular ultrasound (IVUS).

## Methods

### Patient population

This study was retrospectively conducted with patients who received percutaneous coronary intervention (PCI) under IVUS guidance for de novo coronary artery lesions in Bundang CHA medical center between January 1st and December 31st in 2010. Exclusion criteria were patients whose images of IVUS were inadequate for analyses due to severe calcification (with an arc of > 90° of acoustic shadowing, *n* = 28) or poor image (*n* = 7) and patients with severe calcification in whom IVUS catheter could not pass the target lesion before stenting (*n* = 16). Subjects whose IVUS study was done only after stenting (*n* = 49) were also excluded. Among 398 patients who received PCI, 369 lesions of 298 patients were selected and divided into IR positive and negative groups. The diagnosis of ACS was made in accordance with the definition of myocardial infarction revised by the European Society of Cardiology/American College of Cardiology in 2000 [13]. The study protocol was approved by the institutional review board of our hospital.

### IVUS imaging and analyses and definitions of remodeling

Two IVUS systems, a 20-MHz, 2.9 F IVUS system (Eagle Eye, Volcano Corp, Rancho Cordova, CA, USA) and a 40-MHz, 2.6 F IVUS system (Atlantis SR Pro, Boston Scientific Corp, Fremont, CA, USA) were used in this study. The IVUS catheter was advanced >10 mm beyond the lesion and automated pullback was performed to the aorto-ostial junction at a speed of 0.5 mm/s. For every patient, the target lesion site and a proximal reference site were selected for measurement. The target lesion was defined as the site with the smallest minimal lumen diameter (MLD) or ruptured plaque. The proximal reference segment was chosen as the site with the least amount of plaque proximal to the target lesion without any intervening side branch.

**Fig. 1:**
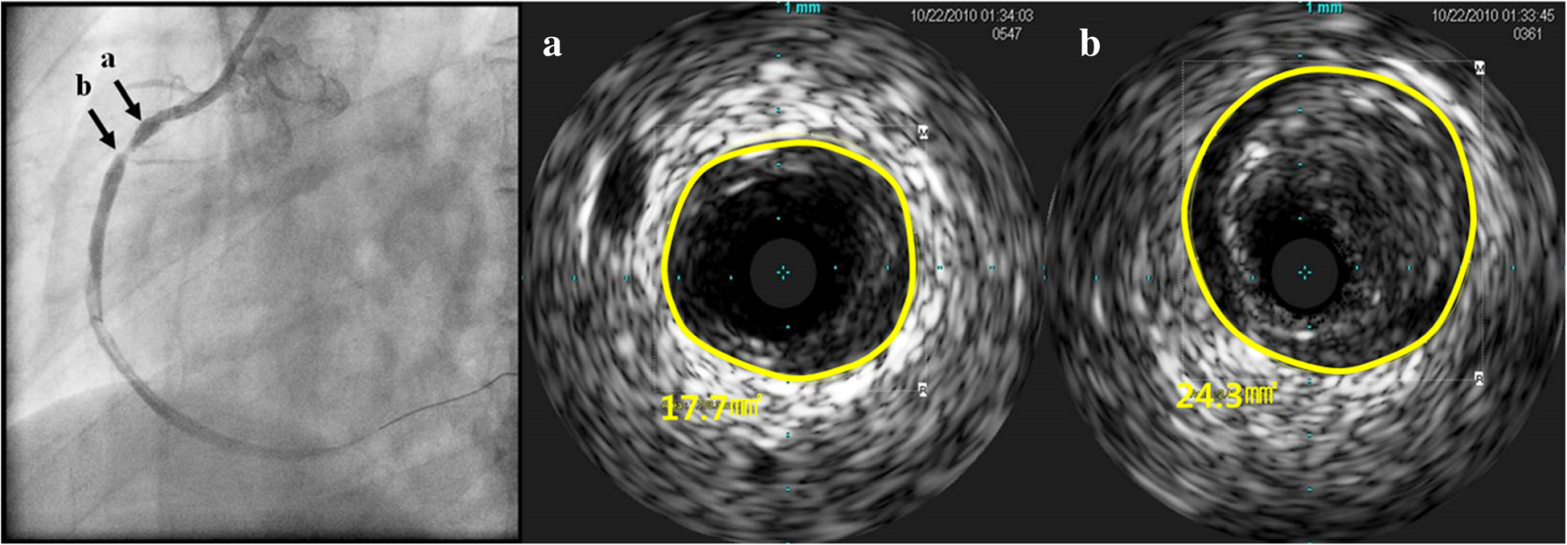
Example case of patient with insulin resistance. Seventy six year-old man who received PCI due to ST elevation myocardial infarction. The positive remodeling of proximal right coronary artery was demonstrated. The remodeling index was 1.373 which was calculated by lesion EEM (arrow **b**, panel **b**, 24.3 mm^2^) divided by reference EEM (arrow **a**, panel **a**, 17.7 mm^2^)

Coronary artery remodeling was defined by comparing the EEM area at the lesion site to the EEM area at the proximal reference site. The remodeling index was defined as the ratio of the EEM area at the lesion site to the EEM area at the proximal reference site in this study (Fig. 1). Patterns of arterial remodeling were classified into three categories; positive remodeling was defined as a remodeling index >1.05; intermediate remodeling as a remodeling index between 0.95 and 1.05; negative remodeling as a remodeling index <0.95 [3, 14].

### Classification of metabolic syndrome and insulin resistance

The status of IR was measured using HOMA-IR, with the following formula; HOMA-IR = fasting insulin (μIU/mL) × fasting glucose (mmol/L)/22*.*5. The presence of MetS was defined by the NCEP ATP III guidelines [15]. MetS scores, ranged from 0 to 5 points, were calculated according to the number of MetS components. Patients whose values exceeded 2.5 of HOMA-IR were considered as an IR positive [16].

### Intra-observer and inter-observer variabilities of IVUS analysis

Two expert operators blinded to the clinical presentation analyzed the IVUS images, and parameters for analysis were presented as the means of both measured parameters. Quantitative measurements of the IVUS images of EEM were reanalyzed at least 1 month apart and used another off-line IVUS analysis system. The correlation coefficient obtained from linear regression analysis and the percent error obtained by calculating the absolute difference divided by the initial measurements [17]. In our institution, the intra-observer correlation coefficient and percent error for EEM were 0.97 and 4.2 ± 4.6 %, respectively, and the inter-observer correlation coefficient and percent error for EEM were 0.95 and 5.2 ± 5.3 %, respectively.

### Statistical analyses

Statistical analyses were performed using the SPSS 19.0 version. Quantitative data was presented as mean ± SD and compared by Student’s *t*-test or Fisher’s exact test when at least 25 % of values showed an expected frequency <5. One-way ANOVA with the multiple comparisons between the remodeling groups was done with post hoc Tamhane test. *p* < 0.05 was considered to be significant. To measure the strength of association between two continuous variables, Pearson correlation analysis was used. Using an epidemiological approach, multivariable linear regression analysis was performed to identify independent predictors of remodeling index by means of a backward stepwise model. Variables associated with remodeling index with a *p*-value less than 0.05 in the univariate analysis or clinically relevant variables were entered into the multivariable model.

## Results

### Baseline characteristics

A total of 298 patients were included in the study and divided into patients with HOMA-IR positive group (130 patients, 43.6 %) and HOMA-IR negative group (168 patients, 56.4 %). Patient’s clinical and laboratory characteristics according to HOMA-IR groups are shown in Table 1. The mean age was 62.6 ± 11.2 years and 67.8 % were men. There were no significant differences in age, gender, smoking history, previous history of MI, CVA and percentage of ACS between the HOMA-IR positive group and negative group. The components of MetS (BMI, hypertension, diabetes, triglycerides and HDL-cholesterol) and MetS score were significantly different between the groups. In laboratory tests, there were no significant differences in hsCRP between the groups but NT-proBNP and HbA1C were significantly higher in the HOMA-IR positive group.

**Table 1:** Baseline characteristics of study population

Characteristics	Total (*n* = 298)	HOMA negative (*n* = 168)	HOMA positive (*n* = 130)	*p*-value
Age (years)	62.6 ± 11.2	62.0 ± 10.8	63.2 ± 11.6	0.357
Male	202 (67.8)	114 (67.9)	88 (67.7)	0.976
Waist Circumference (cm)	89.6 ± 8.2	88.6 ± 8.9	91.2 ± 6.8	0.075
BMI (Kg/m^2^)	24.8 ± 3.1	24.2 ± 3.0	25.5 ± 3.0	<0.001
Hypertension	189 (63.4)	93 (55.4)	96 (73.8)	0.001
Current smoker	141 (47.3)	80 (47.6)	61 (46.9)	0.905
Diabetes Mellitus	89 (29.9)	35 (20.8)	54 (41.5)	<0.001
Previous MI	16 (5.4)	9 (5.4)	7 (5.4)	0.913
Previous CVA	11 (3.7)	7 (4.2)	4 (3.1)	0.554
Acute coronary syndrome	148 (49.7)	78 (46.4)	70 (53.8)	0.204
Number of diseased vessels				0.365
One	105 (35.2)	65 (38.7)	40 (30.8)	
Two	101 (33.9)	54 (32.1)	47 (36.2)	
Three	92 (30.9)	49 (29.2)	43 (33.1)	
Metabolic syndrome score	2.13 ± 1.14	1.74 ± 1.09	2.63 ± 1.00	<0.001
Total cholesterol (mg/dL)	178.9 ± 46.4	177.6 ± 46.7	180.7 ± 46.3	0.564
Triglycerides (mg/dL)	144.5 ± 90.5	134.4 ± 84.2	157.5 ± 96.9	0.029
HDL-C (mg/dL)	42.1 ± 10.2	43.5 ± 10.6	40.3 ± 9.3	0.006
LDL-C (mg/dL)	105.5 ± 39.4	106.0 ± 1.5	104.7 ± 36.5	0.772
HbA1C (%)	6.56 ± 1.33	6.28 ± 1.10	6.95 ± 1.51	<0.001
NT-proBNP (pg/ml)	1233.4 ± 5065.4	562.3 ± 1356.7	2153.7 ± 7562.8	0.043
hsCRP (mg/dl)	0.53 ± 1.18	0.47 ± 1.02	0.61 ± 1.36	0.336
LV Ejection fraction (%)	55.7 ± 12.9	56.1 ± 12.4	55.1 ± 13.5	0.494

Values are presented as mean ± SD or number (%)

*MetS* metabolic syndrome, *BMI* body mass index, *MI* myocardial infarction, *CVA* cerebrovascular accident, *NT-proBNP* N-terminal pro-brain natriuretic peptide, *LV* left ventricle

### Relation between remodeling index and IR

**Fig. 2:**
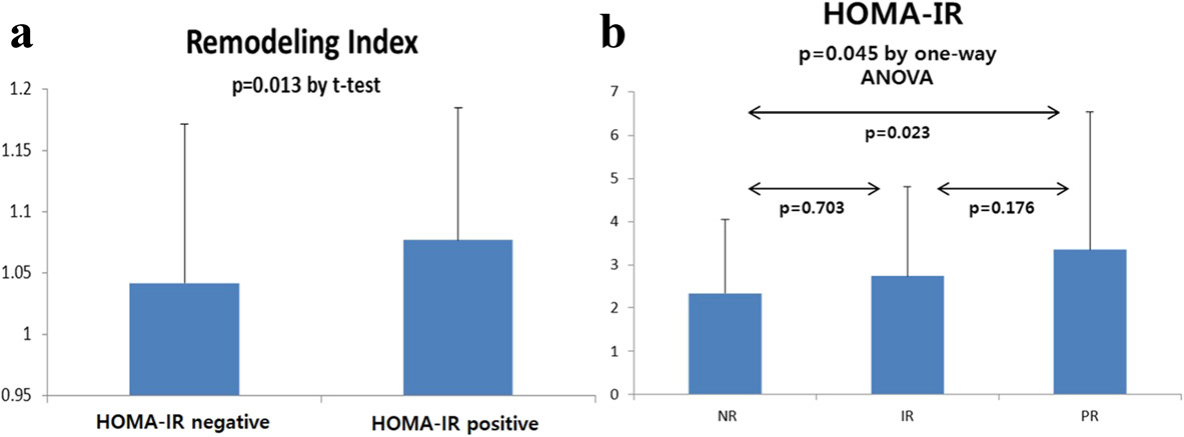
**a**. Remodeling index was significantly higher in the HOMA-IR positive group compared with the negative group (HOMA-IR positive vs. negative: 1.074 ± 0.109 vs. 1.042 ± 0.131, *p* = 0.013). **b**. Relation of remodeling group with HOMA-IR. The level of HOMA-IR is positively correlated with remodeling group (*p* = 0.045 by analyses of one-way ANOVA; NR vs. IR, *p* = 0.703; NR vs. PR, *p* = 0.023; IR vs. PR, *p* = 0.176 by post hoc test). (NR; negative remodeling, IR; intermediate remodeling, PR; positive remodeling)

Comparison of angiographic and IVUS parameters between HOMA-IR positive and negative by lesion were presented in Table 2. A total of 369 lesions were analyzed (161 lesions in HOMA-IR positive and 208 lesions in HOMA-IR negative). Remodeling index was significantly higher in the HOMA-IR positive group compared with the negative group (HOMA-IR positive vs. negative: 1.074 ± 0.109 vs. 1.042 ± 0.131, *p* = 0.013) (Fig. 2a). When the patients were categorized into three groups of positive, intermediate and negative remodeling, there was a tendency that the HOMA-IR positive group had more patients with PR and the HOMA-IR negative group had more patients with NR, but the difference was not statistically significant (*p* = 0.057) (Table 2). Pearson correlation analysis showed that there was a significant positive correlation between remodeling index and HOMA-IR (correlation coefficient = 0.170, *p* = 0.010). The comparison of metabolic indexes among 3 remodeling groups is presented in Table 3. In this analysis, one culprit lesion per patient has been analyzed. There are statistically significant difference of HOMA-IR among remodeling groups (*p* = 0.045) (Fig. 2b). The level of hsCRP was also different among remodeling groups (*p* = 0.045). MetS score showed a similar tendency but there are no statistical significance (*p* = 0.051) (Table 3).

**Table 2:** Comparison of IVUS parameters between HOMA-IR negative and HOMA-IR positive groups

Characteristics	Total *n* = 369 lesions	HOMA-IR negative *n* = 208 lesions	HOMA-IR positive *n* = 161 lesions	*P*-value
Reference EEM area (mm^2^)	14.96 ± 5.27	15.05 ± 5.68	14.84 ± 4.72	0.711
Lesion EEM area (mm^2^)	15.05 ± 5.00	15.06 ± 5.14	15.04 ± 4.82	0.977
Lesions per patient	1.23 ± 0.47	1.24 ± 0.47	1.22 ± 0.47	0.703
Remodeling index	1.056 ± 0.123	1.042 ± 0.131	1.074 ± 0.109	0.013
Remodeling patterns				0.057
Positive remodeling	185 (50.1)	97 (46.6)	88 (54.7)	
Intermediate remodeling	124 (33.6)	69 (33.2)	55 (34.2)	
Negative remodeling	60 (16.3)	42 (20.2)	18 (11.2)	

Values are presented as mean ± SD or number (%)

*IVUS* intravascular ultrasound, *MetS* metabolic syndrome, *EEM* external elastic membrane

**Table 3:** Comparison of metabolic indexes by remodeling patterns

Characteristics	Negative remodeling	Intermediate remodeling	Positive remodeling	*P*-value
	*n* = 50	*n* = 95	*n* = 153	
Age (years)	62.18 ± 10.25	63.38 ± 10.57	62.18 ± 11.73	0.688
Male	40 (66.7)	81 (65.3)	131 (70.8)	0.716
Waist Circumference (cm)	88.5 ± 7.3	89.5 ± 6.6	90.3 ± 9.5	0.576
BMI (Kg/m^2^)	24.8 ± 2.7	24.3 ± 2.8	25.1 ± 3.3	0.106
Total cholesterol (mg/dL)	178.0 ± 48.2	176.9 ± 43.5	180.5 ± 47.9	0.825
Triglycerides (mg/dL)	138.0 ± 82.4	145.1 ± 93.6	146.2 ± 91.6	0.854
HDL-C (mg/dL)	43.3 ± 10.3	42.5 ± 10.0	41.5 ± 10.3	0.528
LDL-C (mg/dL)	100.6 ± 38.8	104.3 ± 37.4	107.8 ± 40.8	0.504
HbA1C (%)	6.56 ± 1.54	6.39 ± 1.09	6.67 ± 1.39	0.309
hsCRP (mg/dl)	0.16 ± 0.27	0.51 ± 1.02	0.67 ± 1.41	0.045
HOMA-IR	2.38 ± 1.80	2.75 ± 2.26	3.55 ± 3.59	0.045
MetS Score	1.78 ± 0.97	2.16 ± 1.14	2.23 ± 1.17	0.051

Values are presented as mean ± SD or number (%)

*BMI* body mass index, *MetS* metabolic syndrome, *hsCRP* high sensitivity C-reactive protein

### Independent factors affecting remodeling index

The common cardiovascular risk factors and metabolic factors were tested as potential predictors of remodeling index (dependent variable) in a regression analysis. Univariate linear regression analysis revealed that presence of MetS and HOMA-IR were independently associated with an increase in remodeling index. Other clinical risk factors (age, sex, smoking history, hsCRP, LDL-C) except components of MetS were not associated with the remodeling index.

Multivariate analysis was performed to identify independent factors affecting remodeling index. Multivariate analysis with linear regression analysis by age, sex, smoking, the presence of MetS and HOMA-IR was performed. Multivariate analysis showed only HOMA-IR was an independent predictor of remodeling index (*r* = 0.166, *p* = 0.018) (Table 4).

**Table 4:** Multivariate analysis for independent factors of remodeling index

Characteristics	Standardized regression coefficient	95% confidence interval		*p*-value
		Lower	Upper	
Age	0.034	−0.001	0.002	0.652
Sex	−0.030	−0.050	0.034	0.706
Smoking	0.044	−0.021	0.038	0.563
MetS	0.045	−0.024	0.047	0.515
hsCRP	0.097	−0.004	0.027	0.157
HOMA-IR	0.166	0.001	0.003	0.018

*MetS* metabolic syndrome, *hsCRP* high sensitivity C-reactive protein

## Discussion

### Positive remodeling and vulnerable plaque

The PR may attenuate the encroachment of the plaque into the lumen, thereby maintaining the lumen area, thus it was thought of arterial enlargement as a beneficial response and negative remodeling as a harmful response to atherosclerotic plaque formation. However, histopathological studies clearly demonstrated that PR is associated with infiltration of inflammatory cells, expression of pro-inflammatory cytokines, and increased protease activity [4, 18].

Moreover, a recently developed virtual histology (VH)-IVUS have facilitated accurate in vivo analysis of coronary plaque and showed that PR is associated with a greater plaque volume and a greater necrotic core [19–21]. Therefore, large plaque burden with increased vessel diameter (PR) determined by IVUS study would be a significant risk factor for rupture of coronary plaques. A recent study of serial IVUS and optical coherence tomography (OCT) showed that positive arterial remodeling was related to thinning change of the fibrous cap [22]. Therefore, it is clear that PR is associated with vulnerable plaque and progression of atherosclerosis.

Vulnerability of coronary atherosclerotic plaques plays a significant role in the occurrence of ACS. It still remains unclear what morphological features will best predict plaque rupture and which diagnostic technologies would reliably predict the pathological and clinical courses of a vulnerable plaque. It also remains unclear what treatment would improve or change the characteristics of coronary plaques. Several study focused the vulnerability and plaque regression [23, 24]. Therefore, early detection of vulnerable plaque before rupture is an important clinical goal for the prevention of catastrophic events like ACS or sudden death and can be a guide for an adjunctive medical or device-based treatment plan.

### Insulin resistance and coronary artery remodeling

Diabetes mellitus is known as a high risk factor for ACS or coronary artery stenosis even though PR is not frequently observed in them [5, 6]. Moreover, underlying pathophysiological determinants that induce negative remodeling in diabetes remain unclear. The IR has been thought as a main cause of type 2 diabetes and MetS. Emerging evidences support a direct proatherogenic effect of IR on the coronary arteries and the advances of understanding molecular mechanisms for atherosclerosis support these findings [9, 25].

Aggravation of IR may be atherogenic via several mechanisms independent of hyperglycemia [26]. Moreover, hyperinsulinemia and IR probably have several atherogenic effects, including the promotion of inflammation and endothelial dysfunction. There are some reports that patients with hyperinsulinemia may have increased plaque vulnerability even before the onset of DM [27] and the larger visceral adipose tissue area is associated with the vulnerable characteristics of coronary plaques in patients without DM, but not in patients with DM [28]. Therefore, it can be inferred IR and abdominal obesity may be a significant cardio-metabolic risk factor that is associated with plaque vulnerability before the development of DM [28]. IR assessed by the HOMA index during the acute phase of the first anterior ST segment elevation MI in patients without diabetes treated by primary PCI is independently associated with poorer myocardial reperfusion, impaired coronary microcirculatory function and potentially with larger final infarct size [29]. Moreover, in patients with MetS, a two- to three-fold increased risk of CAD and cardiovascular mortality has been reported [30–32]. So, the evaluation of IR is very important in the patients with coronary artery disease even though they have no history of DM.

Several studies evaluated the poor outcome of coronary artery disease in the patients of MetS or IR state, but little is known about correlations between IR and coronary artery remodeling associated with coronary plaques. This study focused to demonstrate the coronary artery remodeling and IR. In the present study, remodeling index was significantly higher in the HOMA-IR positive group compared with negative group, and positive remodeling was more common in the HOMA-IR positive group. As mentioned above, positively remodeled vessels with greater plaque volume means increased lipid-rich components. Therefore, our findings proposed that increased remodeling index in the patients with IR are relevant to plaque vulnerability, resulting in increased coronary events such as plaque rupture. Our results are supported by the findings of previous tissue characterization-IVUS studies showing that plaque vulnerability is increased in patients with IR [33]. Finally, based on our findings, it can be inferred that the patient with IR would have more positively remodeled plaques than the patient without IR.

There are several limitations which need to be acknowledged and addressed regarding this study. Firstly, this study was a retrospective, cross-sectional study and follow-up data were not available. Therefore, these results need to be validated by further studies with larger samples. Secondly, severely calcified arteries, other complex lesions and severely stenotic lesions, which might show negative remodeling, were excluded from this study due to limited assessment of coronary morphological disturbances by IVUS. Therefore, the results of this study might represent only a selected group of patients. This could be the cause of the relatively large number of PR patterns in our study.

## Conclusions

In conclusion, increased IR estimated by HOMA-IR was significantly associated with higher remodeling index and positive coronary artery remodeling. The evaluation of IR is important in the patients with coronary artery disease even though they have no history of DM. Further studies regarding effects of insulin resistance on coronary artery remodeling will be needed to validate the results of this study and to provide better understanding about the natural history of atherosclerosis of coronary arteries.

## Abbreviations

ACS: Acute coronary syndrome
BMI: Body mass index
CVA: Cerebrovascular accident
EEM: External elastic membrane
HDL: High-density lipoprotein
HOMA-IR: Homeostasis model assessment of insulin resistance
hsCRP: High sensitivity C-reactive protein
IR: Insulin resistance
IVUS: Intravascular ultrasound
LDL: Low-density lipoprotein
LV: Left ventricle
MetS: Metabolic syndrome
MI: Myocardial infarction
MLD: Minimal lumen diameter
NR: Negative remodeling
NT-proBNP: N-terminal pro-brain natriuretic peptide
PCI: Percutaneous coronary intervention
PR: Positive remodeling
